# Insomnia, and Other Disorders of Sleep

**Published:** 1885-11

**Authors:** 


INSOMNIA AND OTHER DISORDERS OF SLEEP. By Henry M. Lyman, A. M., M. D., Professor of Physiology and of Diseases of the Nervous System in Rush Medical College; Professor of Theory and Practice of Medicine in the Woman's Medical College; and Physician to the Presbyterian Hospital, Chicago, Ill. Chicago: W. T. Keener, 96 Washington street. 1885.

Passing lightly over the poetic emanations of the preface, one desperate plunge takes the reader into the depths of thought to learn that "natural sleep is that condition of physiological repose in which the molecular movements of the brain are no longer fully and clearly projected upon the field of consciousness." If he fathoms this, and keeps wide awake, perhaps the rest of the book may reward his efforts to wade through its 229 pages, which are well printed upon good paper by the enterprising publisher.

